# Supplementary material for: Toxoplasma gondii actin filaments are tuned for rapid disassembly and turnover
Source: Nat Commun. 2024 Feb 28;15:1840. doi: 10.1038/s41467-024-46111-3 (PMC10902351; doi:10.1038/s41467-024-46111-3)
Supplement: Supplementary file 1 — Supplementary Information [file 41467_2024_46111_MOESM1_ESM.pdf]

Supplementary Figures

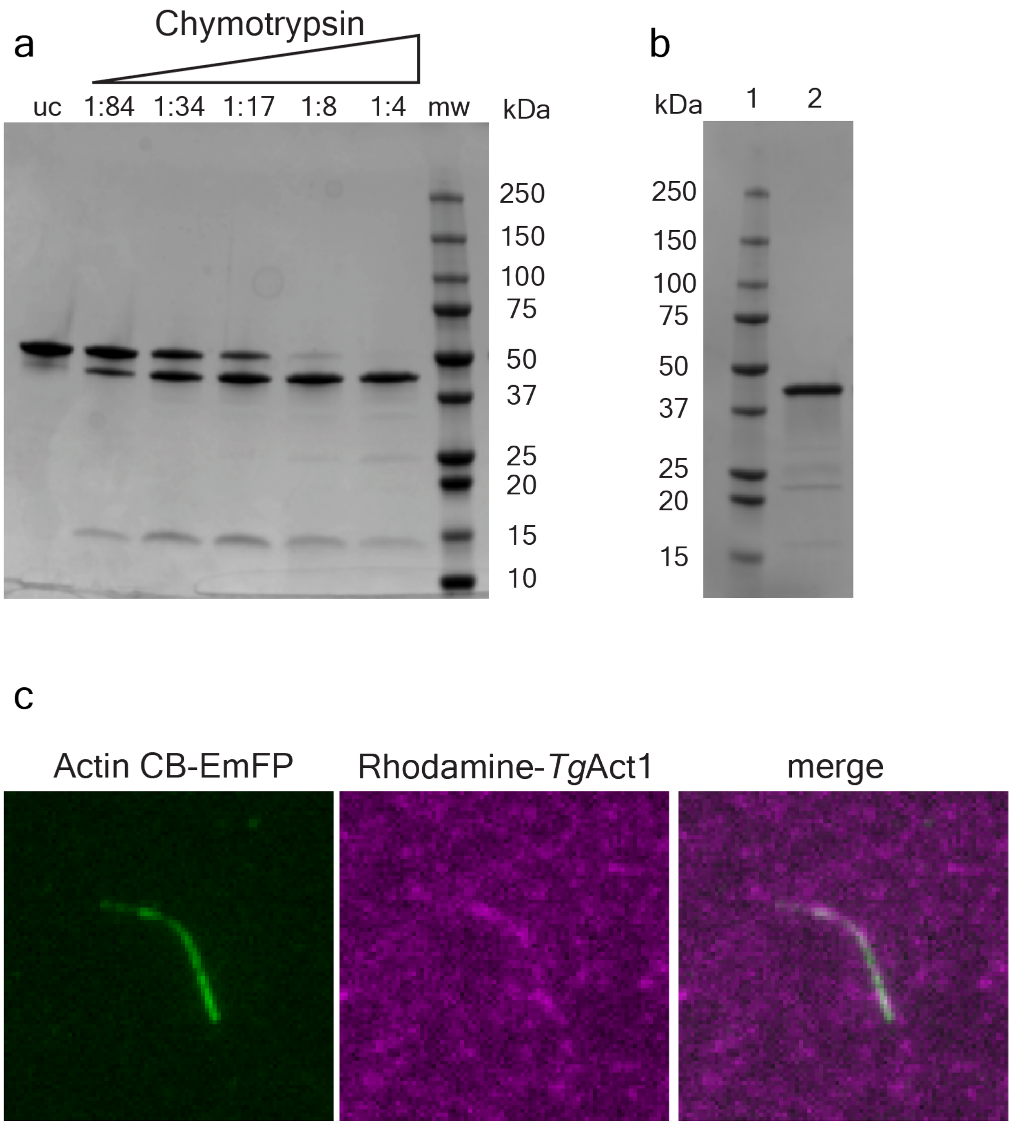

Supplementary Figure 1: Protein preparations and growth of *Tg*Act1 in the presence of labeled Rhodamine actin.

a, Coomassie stained SDS-PAGE showing *Tg*Act1–β-thymosin–HIS incubated with different molar ratios of chymotrypsin:actin for 15 min at 27°C, to determine optimal conditions for removal of β-thymosin–HIS tag. uc: uncut; mw: protein molecular weight marker. b, Coomassie stained SDS-PAGE gel showing (lane 1) protein molecular weight marker and (lane 2) 2 µg purified actin chromobody-EmeraldFP. c, Unlabeled *Tg*Act1 was mixed with Rhodamine-labeled *Tg*Act1 at a ratio of 20:1 (5%) and induced for polymerization for 15 minutes. The resulting filaments were adhered to a flow chamber, washed with an imaging buffer containing actin chromobody-EmeraldFP and imaged. The average percent incorporation (0.5 ± 0.07%, n = 4 filaments) was determined by visualizing the number of Rhodamine positive monomers per filament length. Source data are provided as a Source Data file.

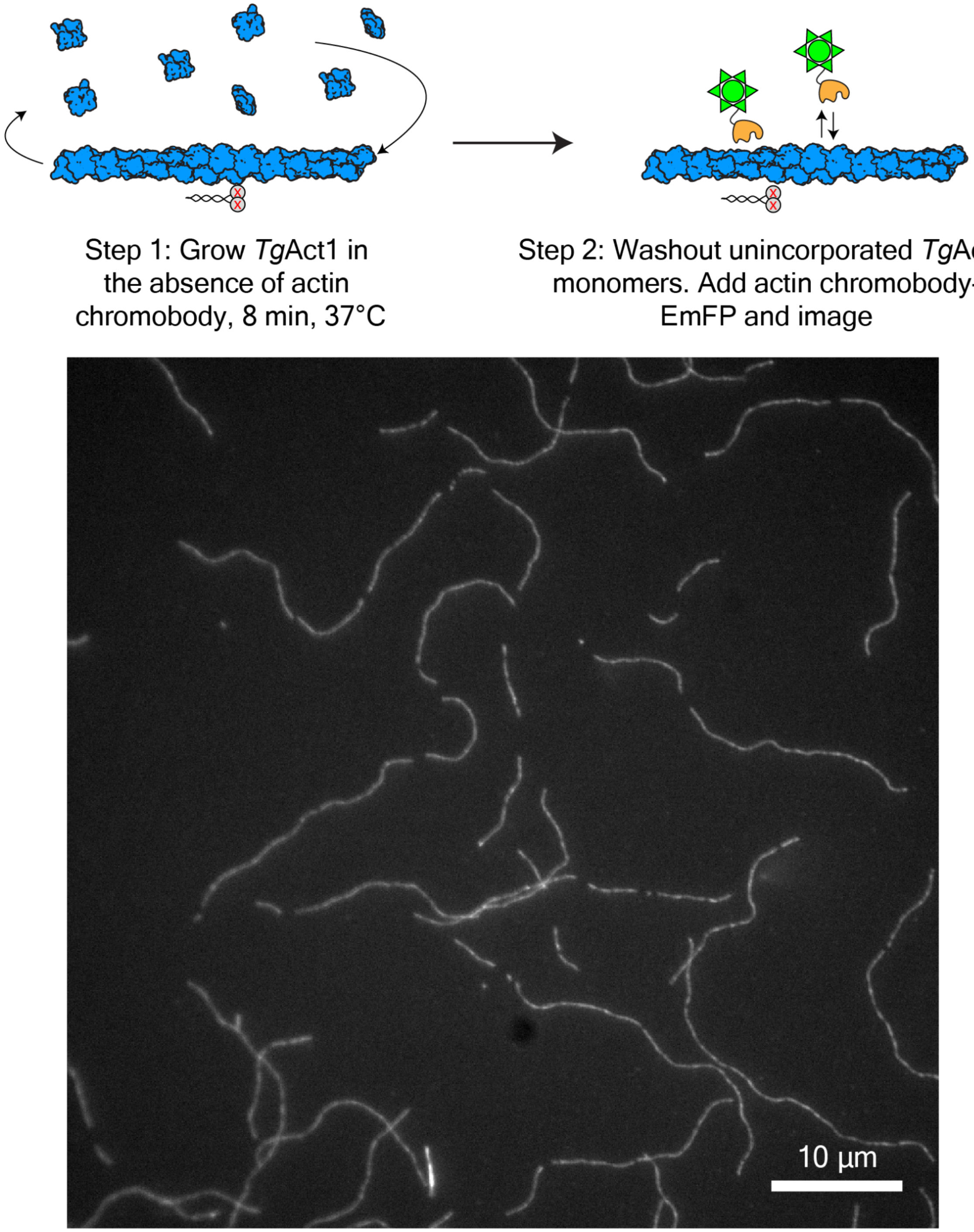

Supplementary Figure 2: Imaging *Tg*Act1 polymerized in the absence of chromobody. *Tg*Act1 was induced for polymerization in a flow chamber in the absence of actin chromobody for 10 min at 37°C. Then, unincorporated *Tg*Act1 monomers were washed out and replaced with a solution containing 50 nM actin chromobody-EmeraldFP and imaged before filaments depolymerized. Polymerization conditions: 25 mM imidazole, pH 7.4, 50 mM KCl, 2.5 mM MgCl2, 1 mM EGTA, 2.5 mM MgATP,10 mM DTT, 0.25% methylcellulose, 2.5 mg/mL BSA, 0.5% Pluronic F-127, oxygen scavenging system (0.13 mg/mL glucose oxidase, 50 µg/mL catalase, and 3 mg/mL glucose), 37°C.

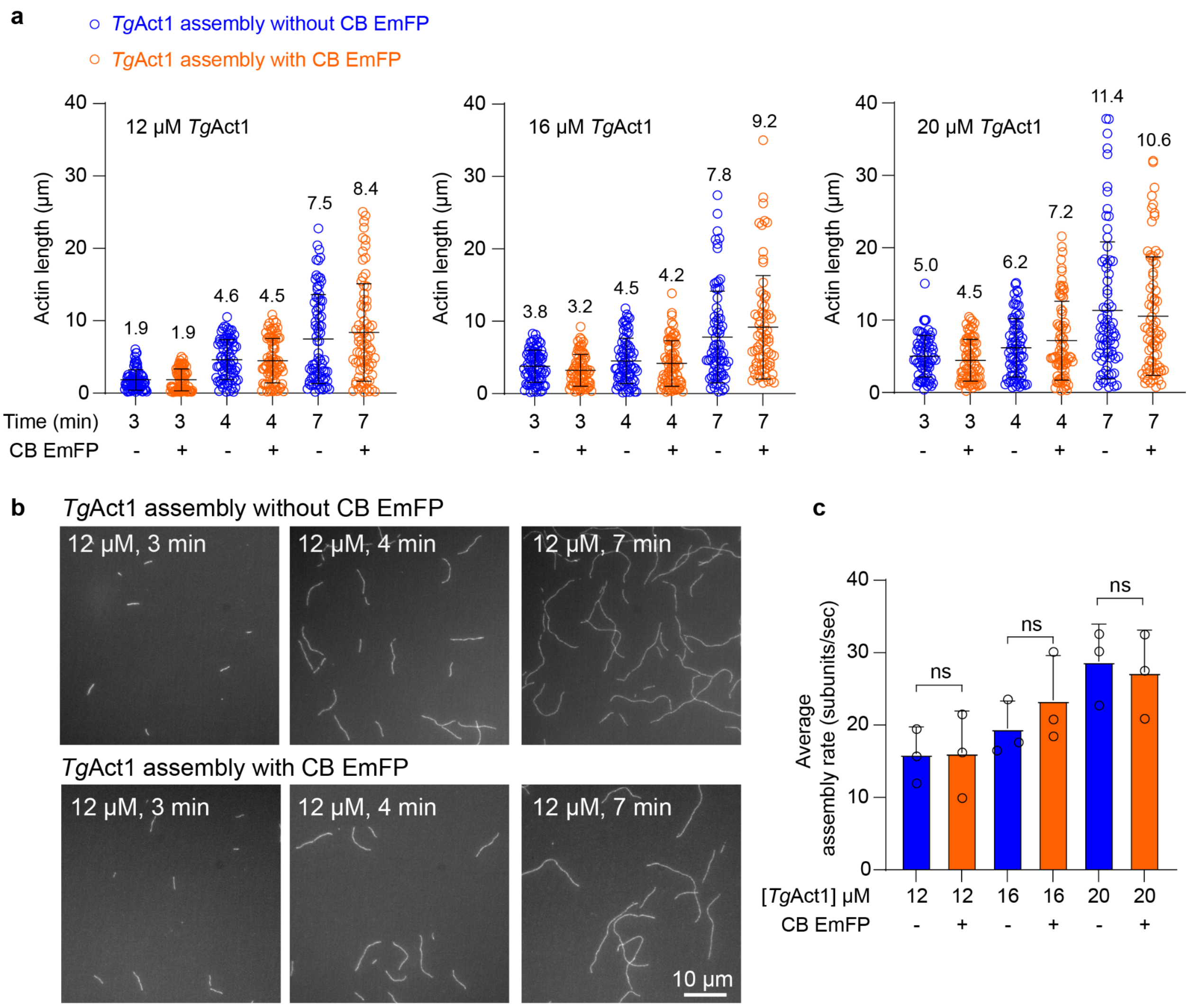

Supplementary Figure 3: The actin chromobody EmFP has no effect on the average assembly rate of *Tg*Act1 filaments.

*Tg*Act1 was induced for polymerization in a flow chamber in the absence or presence of 50 nM actin chromobody (actin CB EmFP). After the indicated time interval, actin monomers were removed to stop further assembly and 50 nM actin chromobody was added to visualize the resulting filaments. a, Comparison of actin length distributions assembled in the absence (blue) or presence of actin CB EmFP (orange) for 3, 4, and 7 minutes at 37°C. Descriptive stastics for actin distributions are shown in Ext. Data Table 1. b, Montage of images showing the assembly of 12 µM *Tg*Act1 in the absence (top) and presence (bottom) of actin CB EmFP over time. c, The average rates of TgAct1 assembly in the absence vs. presence of CB EmFP were determined by comparing the maximum actin lengths over time for each actin concentration. No significant differences in the average assembly rate ± actin CB EmFP were detected by an unpaired two-tailed parametric t-test (12 µM ± actin CB EmFP, $p = 0.969$; 16 µM ± actin CB EmFP, $p = 0.4033$; 20 µM ± actin CB EmFP, $p = 0.752$). Maximum values at each time interval were averaged to generate mean ± SD which was converted to subunits/sec. Mean ± SD; n (12 µM: 16.0 ± 3.8; n=3, 12 µM + actin CB EmFP: 16.2 ± 5.8; n=3, 16 µM: 19.5 ± 3.8; n=3, 16 µM + actin CB EmFP: 23.4 ± 6.2; n=3, 20 µM: 28.8 ± 5.1; n=3, 20 µM + actin CB EmFP: 27.3 ± 5.9;

n=3). Polymerization conditions match those in Figure 1/Table 1. Source data are provided as a Source Data file.

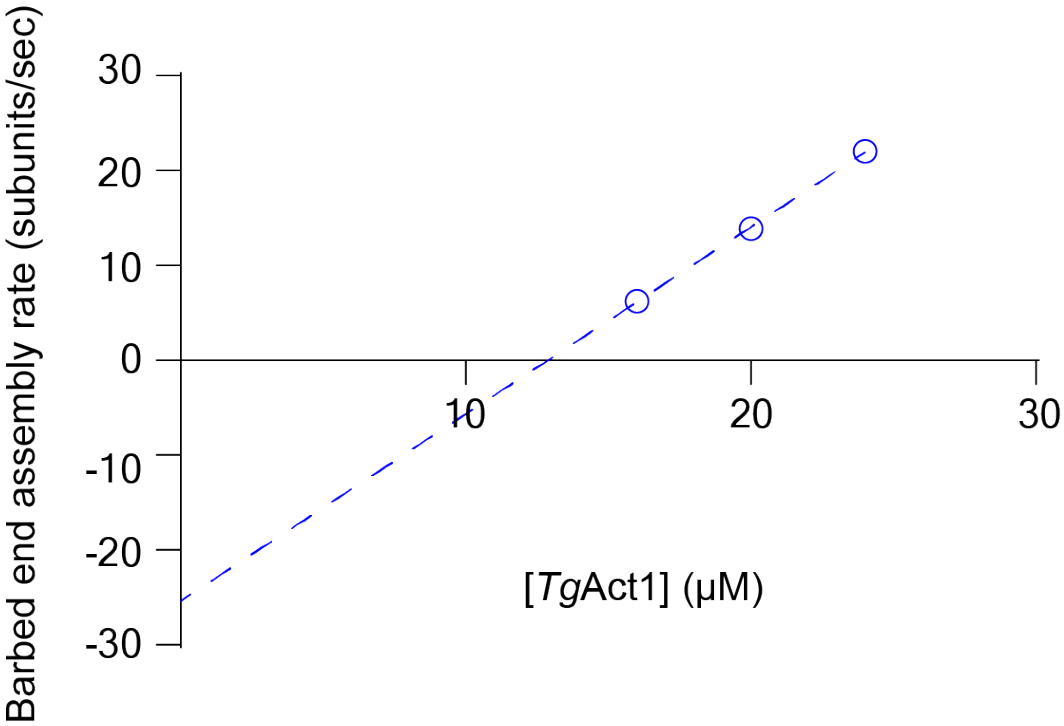

Supplementary Figure 4: Plot of the rate of barbed-end growth in subunits/sec, per actin concentration for *Tg*Act1 in the presence of 0.1 mM AMPPNP.
The critical concentration (C_c) (x-intercept of the fitted line) is 12.9 μM. The dissociation constant (k_-) (y-intercept) is 25.4 sec^{-1} and the association constant (k_+) (slope) is 2.0 sec^{-1} μM^{-1}. Source data are provided as a Source Data file.

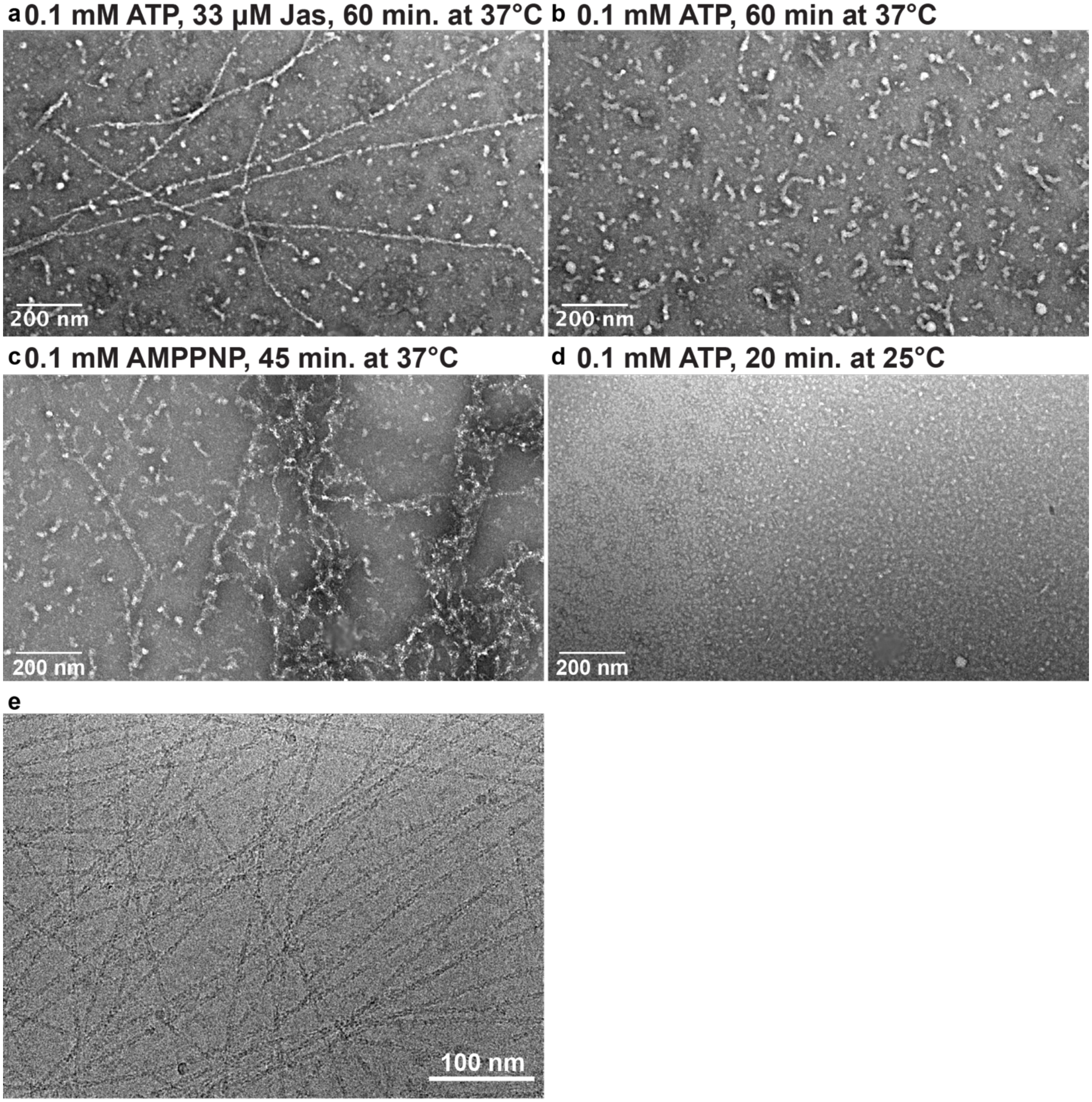

Supplementary Figure 5: Negative stain and cryo electron microscopy of *Tg*Act1 filaments.

a, After 1 hour at 37ºC in polymerization buffer + jasplakinolide, filaments are observed. b, Under the same conditions but without jasplakinolide, no filaments are observed. c, Substitution of AMPPNP for Mg-ATP produces observable filaments. d, No filaments were observed by shortening the incubation time and a decrease in the temperature. For panels a-d, one grid was imaged at a minimum of two separate locations. e, Representative cryo-micrograph from the *Tg*Act1 + jasplakinolide dataset; micrograph count found in Table 2.

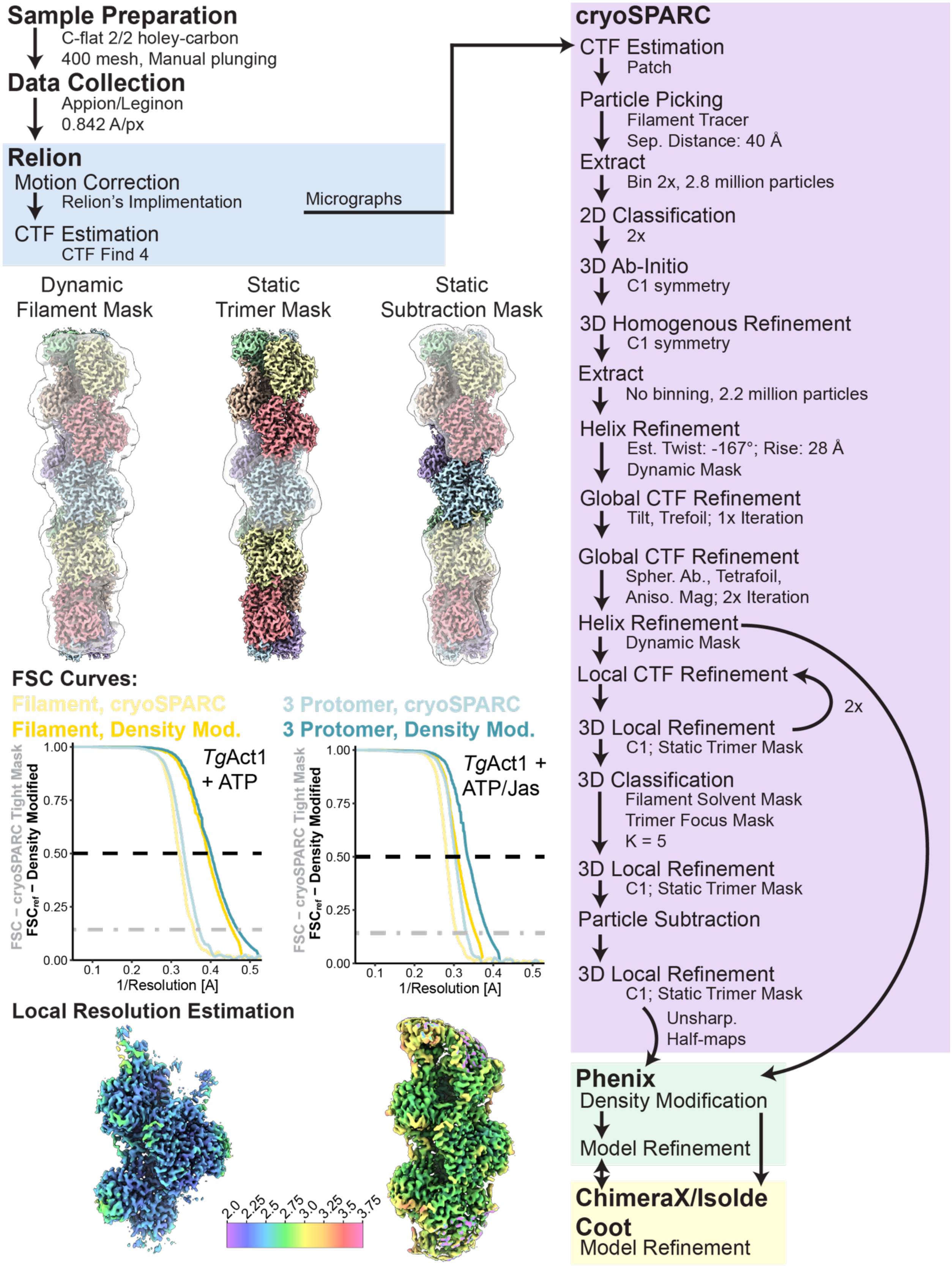

Sample Preparation
C-flat 2/2 holey-carbon
400 mesh, Manual plunging
Data Collection
Appion/Leginon
0.842 A/px
Relion
Motion Correction
Relion's Implimentation
CTF Estimation
CTF Find 4
Micrographs
cryoSPARC
CTF Estimation
Patch
Particle Picking
Filament Tracer
Sep. Distance: 40 Å
Extract
Bin 2x, 2.8 million particles
2D Classification
2x
3D Ab-Initio
C1 symmetry
3D Homogenous Refinement
C1 symmetry
Extract
No binning, 2.2 million particles
Helix Refinement
Est. Twist: -167°; Rise: 28 Å
Dynamic Mask
Global CTF Refinement
Tilt, Trefoil; 1x Iteration
Global CTF Refinement
Spher. Ab., Tetrafoil,
Aniso. Mag; 2x Iteration
Helix Refinement
Dynamic Mask
Local CTF Refinement
2x
3D Local Refinement
C1; Static Trimer Mask
3D Classification
Filament Solvent Mask
Trimer Focus Mask
K = 5
3D Local Refinement
C1; Static Trimer Mask
Particle Subtraction
3D Local Refinement
C1; Static Trimer Mask
Unsharp.
Half-maps
Phenix
Density Modification
Model Refinement
ChimeraX/Isolde
Coot
Model Refinement
Dynamic
Filament Mask
Static
Trimer Mask
Static
Subtraction Mask
FSC Curves:
Filament, cryoSPARC
Filament, Density Mod.
3 Protomer, cryoSPARC
3 Protomer, Density Mod.
TgAct1 + ATP
TgAct1 + ATP/Jas
FSC – cryoSPARC Tight Mask
FSCref – Density Modified
1.00
0.75
0.50
0.25
0.00
0.1
0.2
0.3
0.4
0.5
1/Resolution [A]
Local Resolution Estimation
2.0
2.25
2.5
2.75
3.0
3.25
3.5
3.75

Supplementary Figure 6: Data processing scheme and statistics for cryo electron microscopy datasets.
The flowchart shows the data processing scheme for *Tg*Act1 + 1 mM ATP; a similar scheme without particle subtraction was used for *Tg*Act1 + jasplakinolide. The masks shown were used as described in the flowchart; volume protomers shown in rainbow colors. Fourier shell correlation curves for the helical reconstructions are shown in yellow and curves for the locally-refined, three-protomer volume are shown in blue, shaded based on the software in which they were calculated (cryoSPARC, light colors; Phenix Density Modification, dark colors). Local resolutions estimated in cryoSPARC were mapped onto volumes locally filtered in cryoSPARC; colors represent estimated resolution in scale.

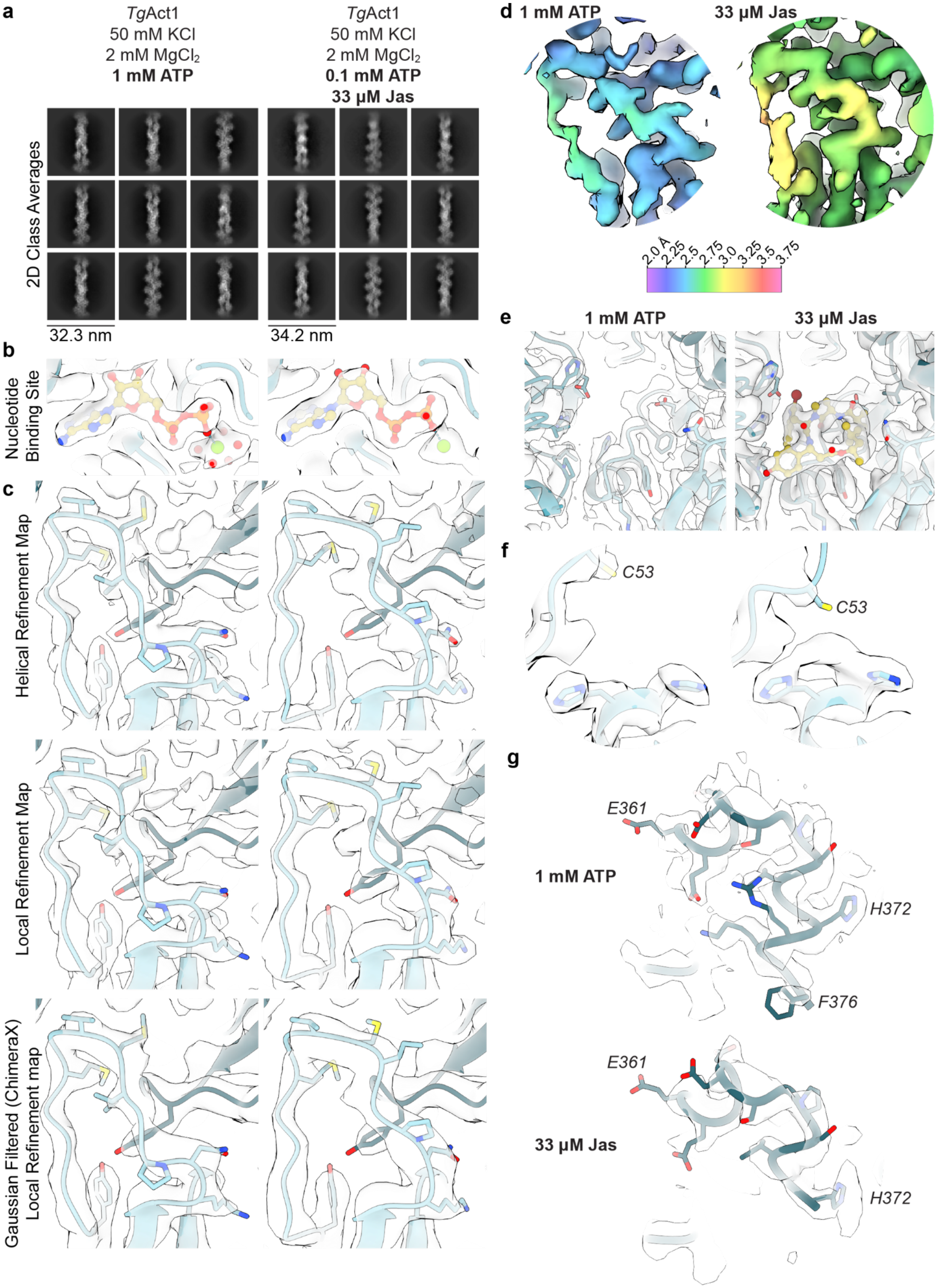

a
TgAct1
50 mM KCl
2 mM MgCl2
1 mM ATP
TgAct1
50 mM KCl
2 mM MgCl2
0.1 mM ATP
33 µM Jas
2D Class Averages
32.3 nm
34.2 nm
b
Nucleotide Binding Site
c
Helical Refinement Map
Local Refinement Map
Gaussian Filtered (ChimeraX) Local Refinement map
d
1 mM ATP
33 µM Jas
2.0 Å
2.25
2.5
2.75
3.0
3.25
3.5
3.75
e
1 mM ATP
33 µM Jas
f
C53
C53
g
E361
1 mM ATP
H372
F376
E361
33 µM Jas
H372

Supplementary Figure 7: Exploration of *Tg*Act1 filament data, maps, and models.
a, Representative two-dimensional class averages from the data sets. b, Map and model from each dataset showing the nucleotide binding site, with the protein in blue and the nucleotide (ADP in both structures) in yellow. c, Volumes from helical refinement (top) and local refinement (middle) maps. The bottom panels show the local refinement map after Gaussian filtering in ChimeraX. All models are the final model for the dataset indicated. d, Local resolution maps of the D-loop for each filament (unstabilized *Tg*Act, left and *Tg*Act1 + jasplakinolide, right). Colors represent estimated resolution in scale. e, Map and model from each dataset showing the jasplakinolide binding site (unstabilized *Tg*Act, left and *Tg*Act1 + jasplakinolide, right). f, View of Cys53 from each dataset showing volume that suggests the cysteine has been oxidized. g, Volumes and models of the C-termini (unstabilized *Tg*Act, top and *Tg*Act1 + jasplakinolide, bottom).

```
  Toxo. Act1      -MADEEVQAL VVDNGSGNVK AGVAGDDAPR AVFPSIVGKP KNPGIMVGME EKDCYVGDEA 59
Plasmo. Act1      -MGEEDVQAL VVDNGSGNVK AGVAGDDAPR SVFPSIVGRP KNPGIMVGME EKDAFVGDEA 59
Chicken Act1      MCDEDETTAL VCDNGSGLVK AGFAGDDAPR AVFPSIVGRP RHQGVMVGMG QKDSYVGDEA 60 (58)
Plasmo. Act2      --MSEEAVAL VVDNGSGMVK SGLAGDDAPK CVFPSIVGRP KMPNIMIGME QKECYVGDEA 58
                     .::. ** * ***** ** :*.******. .*******.* .  .:*:**  :*:.:*****

  Toxo. Act1      QSKRGILTLK YPIEHGIVTN WDDMEKIWHH TFYNELRVAP EEHPVLLTEA PLNPKANRER 119
Plasmo. Act1      QTKRGILTLK YPIEHGIVTN WDDMEKIWHH TFYNELRAAP EEHPVLLTEA PLNPKGNRER 119
Chicken Act1      QSKRGILTLK YPIEHGIITN WDDMEKIWHH TFYNELRVAP EEHPTLLTEA PLNPKANREK 120 (118)
Plasmo. Act2      QNKRGILTLK YPIEHGIVTN WDDMEKIWHH TFYNELRVSP EEHPVLLTEA PLNPKTNREK 118
                  *.******** *******:** ********** *******.:* ****.***** ***** ***.

  Toxo. Act1      MTQIMFETFN VPAMYVAIQA VLSLYSSGRT TGIVLDSGDG VSHTVPIYEG YALPHAIMRL 179
Plasmo. Act1      MTQIMFESFN VPAMYVAIQA VLSLYSSGRT TGIVLDSGDG VSHTVPIYEG YALPHAIMRL 179
Chicken Act1      MTQIMFETFN VPAMYVAIQA VLSLYASGRT TGIVLDSGDG VTHNVPIYEG YALPHAIMRL 180 (178)
Plasmo. Act2      MTQIMFETFD VPAMYVSIQA ILSLYASGRT TGIVLDSGDG VSHTVPIYEG YVLPHAINRI 178
                  *******:*: ******:*** :****:**** ********** *:*.****** *.***** *:

  Toxo. Act1      DLAGRDLTEY MMKILHERGY GFTTSAEKEI VRDIKEKLCY IALDFDEEMK AAEDSSD-IE 238
Plasmo. Act1      DLAGRDLTEY LMKILHERGY GFSTSAEKEI VRDIKEKLCY IALNFDEEMK TSEQSSD-IE 238
Chicken Act1      DLAGRDLTDY LMKILTERGY SFVTTAEREI VRDIKEKLCY VALDFENEMA TAASSSS-LE 239 (237)
Plasmo. Act2      DMAGRDLTYH MMKLFTERGH TFTTTAEREI VRDIKEKLCY IAMDYDEELK RSEEHSDEIE 238
                  *:****** : :**:: ***:  * *:**.** ********** :*:::::*:   : . *. :*

  Toxo. Act1      KSYELPDGNI ITVGNERFRC PEALFQPSFL GKEAAGVHRT TFDSIMKCDV DIRKDLYGNV 298
Plasmo. Act1      KSYELPDGNI ITVGNERFRC PEALFQPSFL GKEAAGIHTT TFNSIKKCDV DIRKDLYGNI 298
Chicken Act1      KSYELPDGQV ITIGNERFRC PETLFQPSFI GMESAGIHET TYNSIMKCDI DIRKDLYANN 299 (297)
Plasmo. Act2      EIYELPDGNL ITVGSERFRC PEALFNPTLI GRECPGLHIT AYQSIMKCDI DIRKELYNNI 298
                  : ******:: **:*.***** **:**:*::: * *..*:* * :::** ***: ****:** *

  Toxo. Act1      VLSGGTTMYE GIGERLTKEL TSLAPSTMKI KVVAPPERKY SVWIGGSILS SLSTFQQMWI 358
Plasmo. Act1      VLSGGTTMYE GIGERLTRDI TTLAPSTMKI KVVAPPERKY SVWIGGSILS SLSTFQQMWI 358
Chicken Act1      VMSGGTTMYP GIADRMQKEI TALAPSTMKI KIIAPPERKY SVWIGGSILA SLSTFQQMWI 359 (357)
Plasmo. Act2      VLSGGTTMYN NIGERLTKEM TNLAPSSMKI KVIAPPERKY SVWIGGSILS SLSTFQQMWI 358
                  *:*******  .*.:*: .:: * ****:*** *::******* *********: **********

  Toxo. Act1      TKEEYDESGP SIVHRKCF 376
Plasmo. Act1      TKEEYDESGP SIVHRKCF 376
Chicken Act1      TKQEYDEAGP SIVHRKCF 377 (375)
Plasmo. Act2      TKEEYEDSGP SIVHRKCF 376
                  **:**:::** ********
```

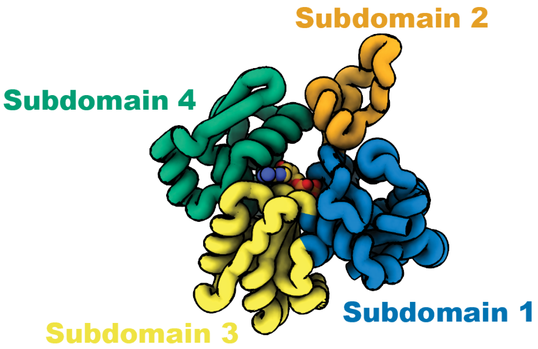

TgAct1 Asn17
DNAse Loop
TgAct1 Tyr54
Hydrophobic Plug
DNAse Loop Pocket

Supplementary Figure 8: Sequence alignment.
Sequence alignment of *Tg*Act1, skeletal actin, *P. falciparum* Act1, and *P. falciparum* Act2. Sequences retrieved from Uniprot (Uniprot IDs: Q8ILW9, P68139, Q8I4X0, P53476) and aligned with the EMBL-EBI portal for MAFFT (v7, https://www.ebi.ac.uk/Tools/msa/mafft/).

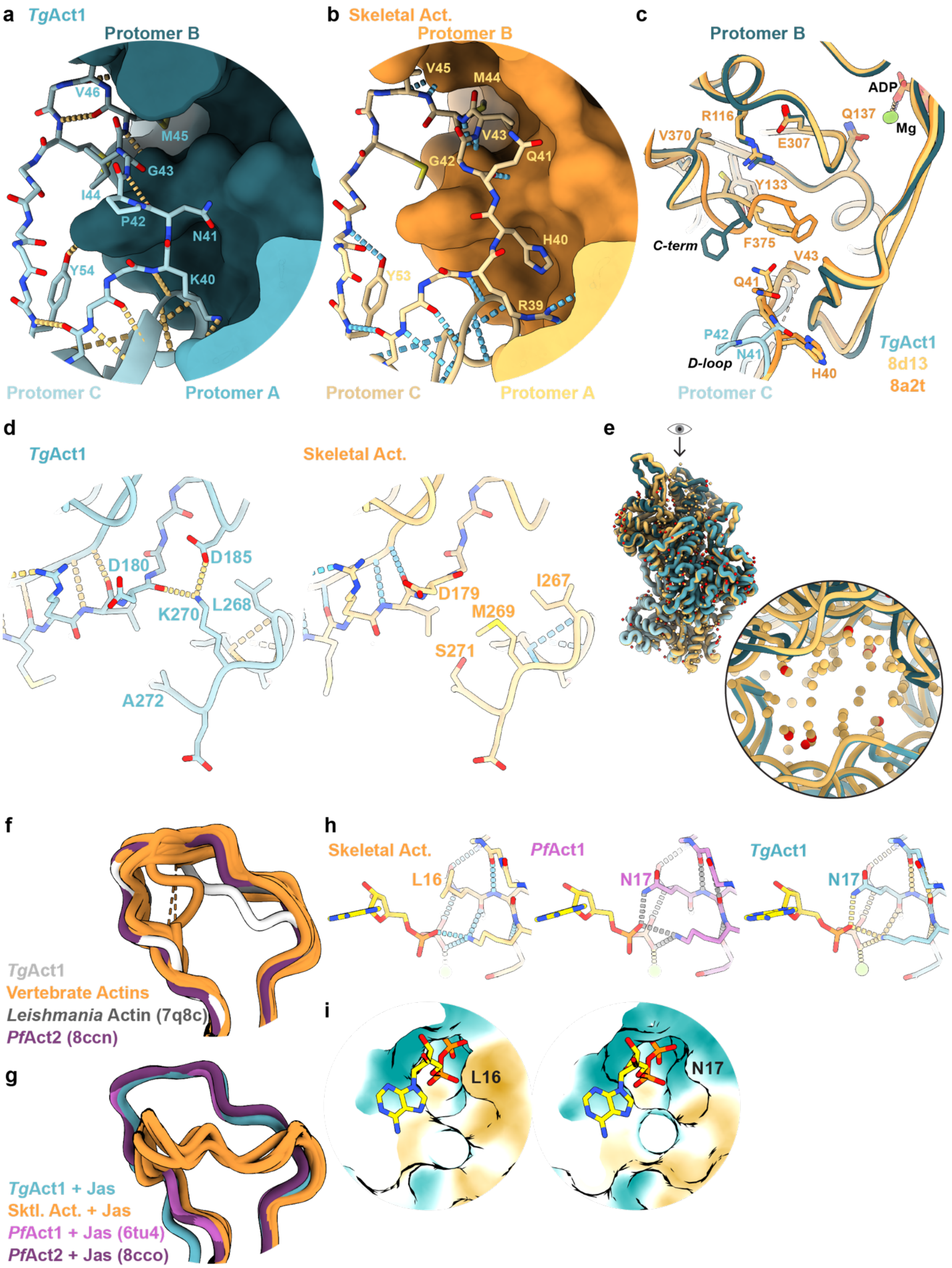

a TgAct1
Protomer B
V46
M45
G43
I44
P42
N41
Y54
K40
Protomer C
Protomer A
b Skeletal Act.
Protomer B
V45
M44
V43
Q41
G42
H40
Y53
R39
Protomer C
Protomer A
c
Protomer B
ADP
Mg
R116
Q137
V370
E307
Y133
C-term
F375
V43
Q41
P42
N41
D-loop
H40
TgAct1
8d13
8a2t
Protomer C
d
TgAct1
D180
D185
K270
L268
A272
Skeletal Act.
D179
I267
M269
S271
e
f
TgAct1
Vertebrate Actins
Leishmania Actin (7q8c)
PfAct2 (8ccn)
g
TgAct1 + Jas
Sktl. Act. + Jas
PfAct1 + Jas (6tu4)
PfAct2 + Jas (8cco)
h
Skeletal Act.
L16
PfAct1
N17
TgAct1
N17
i
L16
N17

Supplementary Figure 9: Actin filament comparisons.
a/b, Hydrogen bonding network of D-loop from unstabilized *Tg*Act1 actin filaments (protein in blue, hydrogen bonds in orange) and skeletal actin filaments (protein in orange, hydrogen bonds in blue). Side chains of amino acids 49-53 (skeletal actin 48-52) have been omitted for clarity. c, Nucleotide sensing and transmission residues from unstabilized *Tg*Act1 (teals) and Mg-ADP skeletal actins (8d13, light orange; 8a2t, dark orange). For clarity, only residue numbers from skeletal actin are shown for Protomer B. d, Hydrogen bonding network of the hydrophobic plug region of unstabilized *Tg*Act1 actin filaments (protein in blue, hydrogen bonds in orange) and skeletal actin filaments (protein in orange, hydrogen bonds in blue). e, Ribbon diagram of three-protomer filament of unstabilized *Tg*Act1 (teals) and skeletal actin (8d13, orange). Waters from *Tg*Act1 shown in red and waters from skeletal actin shown in orange. f, The D-loops from unstabilized *Tg*Act1 (gray), several vertebrate actins (orange, PDB IDs 7bt7, 7r8v, 8a2r, 8a2t, 8d13, 8dmx, 8dmy, 8dnf, 8dnh), *Leishmania* actin (dark gray, PDB ID 7q8c), and *P. falciparum* Act2 (purple, PDB ID 8ccn). g, The D-loops from *Tg*Act1 + jasplakinolide (blue), skeletal actin + jasplakinolide (orange, PDB IDs 5ooc, 5ood, 6t24, 7pm3), *P. falciparum* Act1 + jasplakinolide (magenta, PDB ID 6tu4), and *P. falciparum* Act2 + jasplakinolide (purple, PDB ID 8cco). h, View of Leu16 or Asn17 from the nucleotide binding pocket of skeletal actin filaments (orange, hydrogen bonds in blue), *P. falciparum* Act1 filaments (magenta, hydrogen bonds in grey), and unstabilized *Tg*Act1 (blue, hydrogen bonds in orange). i, Surface representation of the nucleotide binding pocket of skeletal G-actin (PDB ID 1eqy) and *P. falciparum* Act1 G-actin (PDB ID 6i4e) with the nucleotide shown as sticks. Surface colors represent hydrophobicity, with oranges being the most hydrophobic and teals being the most hydrophilic.

Supplementary Data Tables

Condition	Mean ± SD	Max	n
12 µM, 3 min	1.9 ± 1.4	6	78
12 µM, 3 min + actin CB EmFP	1.9 ± 1.5	5	75
12 µM, 4 min	4.6 ± 2.8	10.5	77
12 µM, 4 min + actin CB EmFP	4.5 ± 3.1	10.9	75
12 µM, 7 min	7.5 ± 6.1	22.7	76
12 µM, 7 min + actin CB EmFP	8.4 ± 6.7	25.1	75
16 µM, 3 min	3.8 ± 2.2	8.3	77
16 µM, 3 min + actin CB EmFP	3.2 ± 2.2	9.2	70
16 µM, 4 min	4.5 ± 3.1	11.8	75
16 µM, 4 min + actin CB EmFP	4.2 ± 3.2	13.9	74
16 µM, 7 min	7.8 ± 6.3	27.4	76
16 µM, 7 min + actin CB EmFP	9.2 ± 7.1	35	75
20 µM, 3 min	5.0 ± 2.9	15	69
20 µM, 3 min + actin CB EmFP	4.5 ± 2.9	10.4	75
20 µM, 4 min	6.2 ± 4.0	15.1	75
20 µM, 4 min + actin CB EmFP	7.2 ± 5.4	21.6	75
20 µM, 7 min	11.4 ± 9.5	37.8	76
20 µM, 7 min + actin CB EmFP	10.6 ± 8.2	32	75

Supplementary Table 1: Descriptive statistics for Ext. Data Figure 3a.
Mean, maximum and number of values for actin length distributions assembled in the presence or absence of chromobody (CB-EmFP) for the indicated time period. Source data are provided as a Source Data file.
