# Supplementary material for: Toxoplasma gondii actin filaments are tuned for rapid disassembly and turnover
Source: Nat Commun. 2024 Feb 28;15:1840. doi: 10.1038/s41467-024-46111-3 (PMC10902351; doi:10.1038/s41467-024-46111-3)
Supplement: Supplementary file 2 — Reporting Summary [file 41467_2024_46111_MOESM2_ESM.pdf]

nature portfolio

Corresponding author(s): Aoife Heaslip
Last updated by author(s): Feb 6, 2024

Reporting Summary

Nature Portfolio wishes to improve the reproducibility of the work that we publish. This form provides structure for consistency and transparency in reporting. For further information on Nature Portfolio policies, see our Editorial Policies and the Editorial Policy Checklist.

Statistics

For all statistical analyses, confirm that the following items are present in the figure legend, table legend, main text, or Methods section.

n/a	Confirmed	
☐	☒	The exact sample size (n) for each experimental group/condition, given as a discrete number and unit of measurement
☐	☒	A statement on whether measurements were taken from distinct samples or whether the same sample was measured repeatedly
☐	☒	The statistical test(s) used AND whether they are one- or two-sided<br>*Only common tests should be described solely by name; describe more complex techniques in the Methods section.*
☒	☐	A description of all covariates tested
☒	☐	A description of any assumptions or corrections, such as tests of normality and adjustment for multiple comparisons
☐	☒	A full description of the statistical parameters including central tendency (e.g. means) or other basic estimates (e.g. regression coefficient) AND variation (e.g. standard deviation) or associated estimates of uncertainty (e.g. confidence intervals)
☒	☐	For null hypothesis testing, the test statistic (e.g. F, t, r) with confidence intervals, effect sizes, degrees of freedom and P value noted<br>*Give P values as exact values whenever suitable.*
☒	☐	For Bayesian analysis, information on the choice of priors and Markov chain Monte Carlo settings
☒	☐	For hierarchical and complex designs, identification of the appropriate level for tests and full reporting of outcomes
☒	☐	Estimates of effect sizes (e.g. Cohen's d, Pearson's r), indicating how they were calculated

Our web collection on statistics for biologists contains articles on many of the points above.

Software and code

Policy information about availability of computer code

Data collection	Digital Micrograph (Gatan, 2.10.1282.0), Leginon (v3.3, v3.4, v3.5).
Data analysis	Relion (v4.0), MotionCor2 (v2), CTFFind4 (v4), cryoSPARC (v3 & v4), ISOLDE (v1.5), ChimeraX (v1.5), Coot (v0.9.4.1), Phenix (v1.18 and v1.20), Fiji (v2.1.0), Adobe Illustrator CC (v26.0.1), GraphPad Prism (v10.0.0).

For manuscripts utilizing custom algorithms or software that are central to the research but not yet described in published literature, software must be made available to editors and reviewers. We strongly encourage code deposition in a community repository (e.g. GitHub). See the Nature Portfolio guidelines for submitting code & software for further information.

Data

Policy information about availability of data

All manuscripts must include a data availability statement. This statement should provide the following information, where applicable:

- Accession codes, unique identifiers, or web links for publicly available datasets
- A description of any restrictions on data availability
- For clinical datasets or third party data, please ensure that the statement adheres to our policy

The cryo-EM maps generated for this manuscript are available from the EMDB (https://www.ebi.ac.uk/emdb/) at the accession codes listed in Table 1 of the manuscript (EMDB IDs: EMDB-41583 [https://www.ebi.ac.uk/emdb/EMD-41583], EMDB-41584 [https://www.ebi.ac.uk/emdb/EMD-41584]) The protein models generated for this manuscript are available from the RCSB PDB (https://www.rcsb.org/) at the accession codes listed in Table 1 of the manuscript (PDB IDs: 8TRM

[https://www.rcsb.org/structure/8TRM], 8TRN [https://www.rcsb.org/structure/8TRM]). Previously deposited structures referenced in this manuscript include: 7bt7 [https://www.rcsb.org/structure/7BT7], 7r8v [https://www.rcsb.org/structure/7R8V], 8a2r [https://www.rcsb.org/structure/8A2R], 8a2t [https://www.rcsb.org/structure/8A2T], 8d13 [https://www.rcsb.org/structure/8D13], 8dmx [https://www.rcsb.org/structure/8DMX], 8dmy [https://www.rcsb.org/structure/8DMY], 8dnf [https://www.rcsb.org/structure/8DNF], 8dnh [https://www.rcsb.org/structure/8DNH], 7q8c [https://www.rcsb.org/structure/7Q8C], 8ccn [https://www.rcsb.org/structure/8ccn], 5ooc [https://www.rcsb.org/structure/5OOC], 5ood [https://www.rcsb.org/structure/5OOD], 6t24 [https://www.rcsb.org/structure/6T24], 7pm3 [https://www.rcsb.org/structure/7PM3], 8cco [https://www.rcsb.org/structure/8CCO], 1eqy [https://www.rcsb.org/structure/1EQY], 6i4e [https://www.rcsb.org/structure/6I4E]. Protein sequences were retrieved from Uniprot (https://www.uniprot.org/) at the codes: P53476 [https://www.uniprot.org/uniprotkb/P53476/entry], Q8I4X0 [https://www.uniprot.org/uniprotkb/Q8I4X0/entry], P68139 [https://www.uniprot.org/uniprotkb/P68139/entry], and Q8ILW9 [https://www.uniprot.org/uniprotkb/Q8ILW9/entry]. Source data are provided with this paper.

Human research participants

Policy information about studies involving human research participants and Sex and Gender in Research.

Reporting on sex and gender	There were no human research participants in these studies.
Population characteristics	There were no human research participants in these studies.
Recruitment	There were no human research participants in these studies.
Ethics oversight	There were no human research participants in these studies.

Note that full information on the approval of the study protocol must also be provided in the manuscript.

Field-specific reporting

Please select the one below that is the best fit for your research. If you are not sure, read the appropriate sections before making your selection.

☒ Life sciences ☐ Behavioural & social sciences ☐ Ecological, evolutionary & environmental sciences

For a reference copy of the document with all sections, see nature.com/documents/nr-reporting-summary-flat.pdf

Life sciences study design

All studies must disclose on these points even when the disclosure is negative.

Sample size	No sample size was predetermined. As is standard for screening with negative stain electron microscopy, two to three micrographs from different locations on a single grid were recorded and examined per condition. The number of micrographs collected and particles included in final cryoEM structures are listed in Data Table 1. For actin polymerization microscopy assays, hundreds of filaments from 3 independent protein preps are included in the data as is standard in the field.
Data exclusions	As is standard in cryoEM data analysis, particles that did not contribute to high resolution structure determination were excluded during 2D and 3D classification.
Replication	For electron microscopy experiments, all cryo electron microscopy experiments are conducted as follow up experiments to negative stain screening experiments. All cryo datasets that were collected repeated and built upon the results from the negative stain screening experiments. For actin polymerization assays, three independent protein preps were used in data generation and no differences among preps were found.
Randomization	No grouping was used in this study.
Blinding	No blinding was used in this study as the individual collecting the data also performed the analysis.

Reporting for specific materials, systems and methods

We require information from authors about some types of materials, experimental systems and methods used in many studies. Here, indicate whether each material, system or method listed is relevant to your study. If you are not sure if a list item applies to your research, read the appropriate section before selecting a response.

Materials & experimental systems

n/a	Involved in the study	
☐	☒	Antibodies
☐	☒	Eukaryotic cell lines
☒	☐	Palaeontology and archaeology
☒	☐	Animals and other organisms
☒	☐	Clinical data
☒	☐	Dual use research of concern

Methods

n/a	Involved in the study	
☒	☐	ChIP-seq
☒	☐	Flow cytometry
☒	☐	MRI-based neuroimaging

Antibodies

Antibodies used

Actin Antibody (Gift from Marcus Meissner); Goat anti-rabbit secondary antibody conjugated to IRDye® 680RD (LI-COR, Cat. No. 926-68071)

Validation

Used previously to detect T. gondii actin in western blot assays. Reference:
Whitelaw JA, Latorre-Barragan F, Gras S, Pall GS, Leung JM, Heaslip A, Egarter S, Andenmatten N, Nelson SR, Warshaw DM, Ward GE, Meissner M. Surface attachment, promoted by the actomyosin system of Toxoplasma gondii is important for efficient gliding motility and invasion. BMC Biol. 2017;15(1):1. doi: 10.1186/s12915-016-0343-5. PubMed PMID: 28100223; PMCID: PMC5242020.

Eukaryotic cell lines

Policy information about cell lines and Sex and Gender in Research

Cell line source(s)

Human foreskin fibroblasts (HFFs): Cat. No. SRC-1041, ATCC; T. gondii RH strain: Gift from Dr. Gary Ward, University of Vermont.

Authentication

None of the cell lines were authenticated.

Mycoplasma contamination

Cell lines were not tested for Mycoplasma contamination.

Commonly misidentified lines
(See ICLAC register)

No commonly misidentified lines were used in this study.
