# Supplementary material for: Toxoplasma gondii actin filaments are tuned for rapid disassembly and turnover
Source: Nat Commun. 2024 Feb 28;15:1840. doi: 10.1038/s41467-024-46111-3 (PMC10902351; doi:10.1038/s41467-024-46111-3)
Supplement: Supplementary file 4 — Description of additional supplementary files [file 41467_2024_46111_MOESM4_ESM.pdf]

DESCRIPTION OF ADDITIONAL SUPPLEMENTARY FILES

Supplementary Movie 1: Direct real-time visualization of TgAct1 polymerization in the presence of 16 µM TgAct1 and 25 nM actin chromobody-EmeraldFP. Conditions: 25 mM imidazole, pH 7.4, 50 mM KCl, 2.5 mM MgCl2, 1 mM EGTA, 2.5 mM MgATP,10 mM DTT, 0.25% methylcellulose, 2.5 mg/mL BSA, 0.5% Pluronic F-127, oxygen scavenging system (0.13 mg/mL glucose oxidase, 50 µg/mL catalase, and 3 mg/mL glucose), 37°C. 100x playback, Image width 66 µm.

Supplementary Movie 2: Example of treadmilling TgAct1 filaments in the presence of 16 µM TgAct1 and 25 nM actin chromobodyEmeraldFP. 100x playback, Image width 21 µm.

Supplementary Movie 3: Ribbon morph between an unstabilized TgAct1 filament and skeletal actin filament. Movie morphs from unstabilized TgAct1 filament to the skeletal muscle actin filament (8d13) and returns to the unstabilized TgAct1 filament.

Supplementary Movie 4: Ribbon morph between an unstabilized TgAct1 filament and jasplakinolide-bound TgAct1 filaments. Movie morphs from unstabilized TgAct1 filament to the jasplakinolide-bound TgAct1 and returns to the unstabilized TgAct1 filament. Jasplakinolide shown in yellow for reference.
